# Is Hypovitaminosis D Associated with Stress Perception in the Elderly? A Nationwide Representative Study in Korea

**DOI:** 10.3390/nu8100647

**Published:** 2016-10-19

**Authors:** Mieun Gwon, Young Jin Tak, Yun Jin Kim, Sang Yeoup Lee, Jeong Gyu Lee, Dong Wook Jeong, Yu Hyeon Yi, Seung Hoon Lee, Hye Rim Hwang, Youngin Lee

**Affiliations:** 1 Department of Family Medicine, Pusan National University School of Medicine, Busan 602-739, Korea; mieun0817@naver.com (M.G.); yujkim@pusan.ac.kr (Y.J.K.); eltidine@hanmail.net (J.G.L.); eeugus@hanmail.net (Y.H.Y.); greatseunghun@hanmail.net (S.H.L.); hezera83@naver.com (H.R.H.); ylee23@daum.net (Y.L.); 2 Biomedical Research Institute, Pusan National University Hospital, Busan 602-739, Korea; 3 Medical Education Unit and Medical Research Institute, Pusan National University School of Medicine, Yangsan 626-870, Korea; saylee@pnu.edu; 4 Family Medicine Clinic and Research Institute of Convergence of Biomedical Science and Technology, Pusan National Yangsan Hospital, Yangsan 626-789, Korea; dwjeong75@hanmail.net

**Keywords:** hypovitaminosis D, vitamin D, stress perception, elderly, Korea

## Abstract

Hypovitaminosis D and stress are common problems among the elderly. The aim of this cross-sectional nationally representative study was to evaluate the association between hypovitaminosis D and stress perception using large-scale nationally representative data from the Korea National Health and Nutrition Examination Survey (2012–2013). In our study, a total of 1393 elders (≥65 years old) were included to evaluate the association between hypovitaminosis D and stress perception. Serum 25-hydroxyvitamin D levels were determined using radioimmunoassay, and perceived stress status was assessed by a self-reporting questionnaire. The association between hypovitaminosis D and stress perception according to sex was examined using logistic regression analysis. After multivariate adjustment for sociodemographic and lifestyle factors and comorbidities, hypovitaminosis D was significantly associated with perceived stress (odds ratio, 2.73; 95% confidence interval, 1.10–6.77; *p* = 0.029) among women; however, this association was not significant among men. Hypovitaminosis D was a risk factor for higher stress perception in older Korean women. Even though the role of vitamin D in stress perception is still unclear, we suggest screening for hypovitaminosis D among the elderly.

## 1. Introduction

Hypovitaminosis D is common in elders as a result of a decreased capacity of the skin to produce vitamin D, diminished sun exposure due to reduced outdoor activity, and reduced dietary intake [1]. In the elderly, hypovitaminosis D has been linked to poor health outcomes such as fractures [2], poor physical function [3], sarcopenia [4], pain [5], use of nursing home care [6], mortality [7], and chronic diseases including osteoporosis, diabetes mellitus, cancer, cardiovascular, neurodegenerative, autoimmune, and infectious diseases [8,9,10]. According to increasing experimental evidence, it has been proposed that metabolites of 25-hydroxyvitamin D, hereinafter referred to as 25(OH)D, can cross the blood brain barrier, and 25(OH)D receptors exist in the central nervous system (CNS) [11,12], which suggests that 25(OH)D might be potentially related to brain function [10,12]. For example, depression and decreased cognitive function have been reported in people with hypovitaminosis D [13,14,15,16,17,18,19,20,21,22,23].

Stress can be defined as the response of the body to internal and external threatening factors. Stress produces physiological effects on peripheral organs, starting from the CNS, via hormonal secretion [24]. The hypothalamic–pituitary–adrenal (HPA) axis is hyperactive during stress, and elevated levels of stress hormones (i.e., glucocorticoids and catecholamines) affect the development and progression of several diseases [25]. Chronic stress may cause a variety of diseases such as diabetes mellitus, hypertension, dyslipidemia, cardiovascular diseases, and digestive diseases [25]. Moreover, elevated levels of stress hormones can cause mental problems such as cognitive disorders and depression [25]. In addition, stress is proposed as a major risk factor for suicide. This is especially important in Korea, which has the highest suicide rate among the Organization for Economic Cooperation and Development countries [26,27,28]. Past traumatic experiences cause increased actual vulnerability among elders, as most elders experience physical, psychological, and social changes related with health problems, decreased economic status, and loneliness, all of which cause stress [29]. Furthermore, elders are very vulnerable to stress because they do not have as many resources or social support as the younger population [30].

As mentioned above, the association between hypovitaminosis D and depression has been shown in many studies. Vitamin D has been shown to down-regulate inflammatory mediators, which have been associated with sickness behavior, psychosocial stress, and depression [10,31]. Subjects with depression perceive more stress [32,33]. Therefore, we hypothesized that hypovitaminosis D has a negative effect on brain function, and in the same stress situation, people with hypovitaminosis D are more likely to perceive stress. However, our understanding of the association between hypovitaminosis D and perceived stress status in human and large-scale studies is limited. These amount of converging evidence points to the need to investigate the relationship between hypovitaminosis D and stress perception.

The aim of this study was to evaluate the association between hypovitaminosis D and stress perception in the elderly, after adjusting for multiple confounding factors, using large-scale nationally representative data from the Korean Health and Nutrition Examination Survey (KNHANES) V-VI. Our findings may contribute to the improvement of both screening of psychological stress and treatment with vitamin D supplements.

## 2. Materials and Methods

### 2.1. Study Population

**Figure 1:**
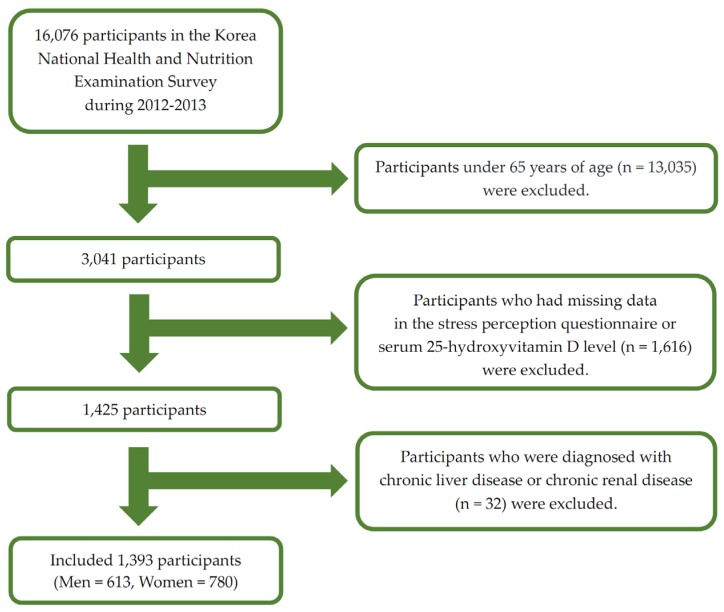
Flow diagram of the study participants.

This nationwide cross-sectional study was based on data from the KNHANES V-VI conducted from 2012 to 2013. The KNHANES was a nationwide survey conducted by the Ministry of Health and Welfare. The KNHANES consisted of three components including a health interview, health examination, and a nutrition survey. A nationally representative sample was selected from the Korean population using household records developed by the 2005 Population and Housing Census in Korea. Twenty households from each district were selected using a stratified, multistage probability cluster sampling system that considers the geographical area, age, and sex of each participant. In the KNHANES V (2012) and VI (2013), 16,076 individuals participated in the examination. In the present study, we included the elderly population (those aged ≥65 years) (Figure 1) with data for important analytic variables such as serum 25(OH)D levels and the stress perception questionnaire. We excluded those diagnosed with liver cirrhosis and chronic renal diseases to eliminate factors affecting 25(OH)D deficiency. Ultimately, 1393 participants were included in the statistical analysis. The institutional review board of the Centers for Disease Control and Prevention in Korea approved the KNHANES. All participants in the survey provided written informed consent (2012-01EXP-01-2C, 2013-07CON-03-4C).

### 2.2. General Characteristics of the Participants

Anthropometric measurements were performed at local community health clinics and centers involved with the health investigation. Anthropometric variables such as height and body weight were measured using a standardized protocol. Body mass index (BMI) was calculated as weight (kg)/height squared (m^2^). Waist circumference was recorded midway between the lower margin of the final rib and the upper margin of the iliac crest to the closest 0.1 cm. Sociodemographic, lifestyle behavior, and physical illness information were collected using a questionnaire. Monthly income, in South Korean currency, was reported and categorized into quartiles according to the equivalent household income as follows: lowest, lower-middle, upper-middle, or highest. We divided the participants into two groups: low-income group (lowest or lower-middle) and high-income group (upper-middle or highest). Based on a self-reported history of cigarette smoking, subjects were classified as follows: current smokers (those who were currently smoking or had smoked more than five packs of cigarettes during their lifetime) and nonsmokers (those who had never smoked or smoked less than five packs of cigarettes during their lifetime). Smoking status was classified according to the National Health and Nutrition Examination Survey (NHANES). Alcohol consumption was defined as consuming at least one alcoholic drink per month. Regular exercise was classified into two categories (“perform” or “do not perform”) based on performing any of the following: intense physical activity for at least 20 min for >3 days/week, moderate physical activity for at least 30 min for >5 days/week, or walking for at least 30 min for >5 days/week. Regular exercise was classified according to the International Physical Activity Questionnaire (IPAQ). Sleep duration to the nearest hour was identified according to the answer to the question: “How much time do you sleep in an ordinary day?” History of comorbidity was evaluated based on the answers (“yes” or “no”) to questions about the diagnosis of hypertension, diabetes mellitus, and dyslipidemia. All participants were requested to maintain their usual dietary habits before assessing dietary intake. Dietary intake was evaluated using the 24-h recall method. Daily energy intake was estimated with the Can-Pro 2.0 nutrient intake assessment software (Korean Nutrition Society, The Korean Nutrition Information Center; Seoul, Korea, 2002).

### 2.3. Serum 25(OH)D Assessment

Blood samples were collected after at least 8 h of fasting time. Blood samples were obtained from each participant during the survey and immediately centrifuged, refrigerated, and transferred into a refrigerator at the Central Testing Institute in Seoul, Korea. Serum 25(OH)D levels were determined by the 25-hydroxyvitamin D 125I radioimmunoassay RIA Kit (DiaSorin Inc., Stillwater, MN, USA) using a γ-counter (1470 Wizard, PerkinElmer, Turku, Finland). The participants were subdivided into the following two groups [34] based on serum 25(OH)D levels to determine the association between hypovitaminosis D and perceived stress status: hypovitaminosis D (25(OH)D level <30 nmol/L) or vitamin D sufficiency (25(OH)D level ≥30 nmol/L).

### 2.4. Perceived Stress Status

Stress perception was assessed using self-reports that involved typical stress identification following the question: “During usual life activities, how much do you feel stressed?” Based on the self-report, perceived stress during daily life was divided on a scale of 1 to 4 (1 = almost no stress, 2 = a little stress, 3 = much stress, 4 = very much stress). The participants were classified into the following two groups: high stress group (score of 3 or 4) and low stress group (score of 1 or 2).

### 2.5. Statistical Analysis

Data from the two surveys (KNHANES 2012 and 2013) were combined. The sampling results were weighted to allow for nationally representative prevalence estimates of the Korean population. The weights were calculated by accounting for the complex survey design, survey non-response, and post-stratification [35]. Therefore, participants were presumed to represent the Korean population after weighting the data. The clinical characteristics of the study population between men and women were compared using the Pearson’s chi-squared tests for categorical variables and generalized linear model for continuous variables. Pearson’s chi-squared tests were used to determine the association between hypovitaminosis D and perceived stress status. Data are presented as estimated mean ± standard error (%; unweighted number) for categorical variables or estimated mean ± standard error for continuous variables. Cramer’s V and partial eta squared were calculated to examine effect sizes. To calculate odds ratios and 95% confidence intervals for the association between hypovitaminosis D and stress perception, we conducted multivariable adjusted logistic regression analysis. Adjusted variables were age; BMI; waist circumference; smoking status; alcohol consumption; physical activity; and history of hypertension, diabetes mellitus s, and dyslipidemia. All analyses were performed using SPSS for Windows (version 18.0; SPSS Inc., Chicago, IL, USA). A *p*-value < 0.05 was considered statistically significant.

## 3. Results

### 3.1. Participant Characteristics

**Figure 2:**
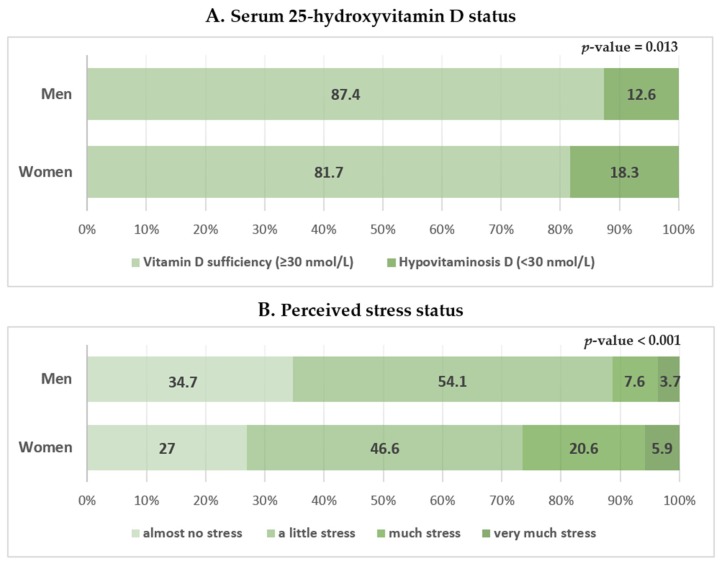
Serum 25-hydroxyvitamin D level and perceived stress status according to sex. Perceived stress status in daily life was assessed using the following question in the self-reported questionnaire: “During usual life activities, how much do you feel stressed?”; 1 = almost no stress, 2 = a little stress, 3 = much stress, 4 = very much stress (high and low stress groups were defined as those with scores of 3 or 4 and 1 or 2, respectively). Data are presented as estimated means (%). *p*-values were obtained by Pearson’s chi-squared using a complex sample design.

The participant characteristics are shown in Table 1. This study included 613 men and 780 women. The mean serum 25(OH)D concentration in men was significantly higher than that in women (47.90 ± 0.85 vs. 45.64 ± 0.92 nmol/L). A higher proportion of women had hypovitaminosis D (<30 nmol/L) than men (18.3% ± 1.7% vs. 12.6% ± 1.8%; Figure 2A). High stress was less frequently reported in men than in women (11.2% ± 1.5% vs. 26.4% ± 2.1%; Figure 2B). Women were older and had a higher BMI than men. Women had a lower household income, energy intake, and regular exercise rate; a higher rate of hypertension, diabetes mellitus, and dyslipidemia; and were less likely to be current smokers or consume alcohol than men.

The characteristics of the study participants according to vitamin D levels for men and women are shown in Table 2 and Table 3, respectively. Men with hypovitaminosis D tended to live in urban areas (88.0% ± 4.4%), be current smokers (34.9% ± 6.6%), and have a lower energy intake (4941 ± 309.11 kJ/day) compared with those with vitamin D sufficiency. Women with hypovitaminosis D were younger (70.69 ± 0.56 years) than those with vitamin D sufficiency.

### 3.2. Association between Hypovitaminosis D and Perceived Stress Status

**Figure 3:**
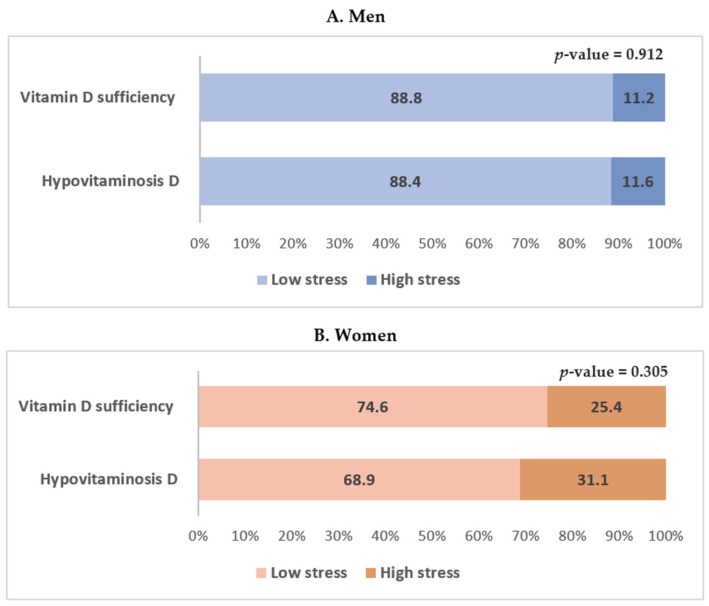
Serum 25-hydroxyvitamin D level according to perceived stress status in men and women. Data are presented as estimated means (%). *p*-values were obtained by Pearson’s chi-squared using a complex sample design.

The serum 25(OH)D level according to perceived stress status in men and women is shown in Figure 3. High perceived stress was more frequently reported in men and women with hypovitaminosis D, but the results were not statistically significant. The odds ratios and 95% confidence intervals of perceived stress status according to vitamin D levels in men and women are shown in Table 4. In the multiple logistic regression analysis, hypovitaminosis D was not significantly associated with perceived stress status in both unadjusted and adjusted models in men (Table 4). In women, hypovitaminosis D was significantly associated with perceived stress status in the fully adjusted model which included age; BMI; waist circumference; smoking status; alcohol consumption; physical activity; and a history of hypertension, diabetes mellitus, and dyslipidemia (Model 3). The odds ratio and 95% confidence intervals for perceived stress status in women with hypovitaminosis D were 2.739 (1.107–6.774) (*p* = 0.029; Table 4).

## 4. Discussion

Hypovitaminosis D and stress are highly prevalent among elders and both have been connected to poor health consequences. Using nationally representative data, we determined the association between hypovitaminosis D and stress perception among Korean elders. To estimate the correlation between hypovitaminosis D on stress perception, we constructed three models to adjust for potential confounders, including age, BMI, waist circumference, smoking status, alcohol consumption, physical activity, and history of chronic diseases. No association was evident between hypovitaminosis D and perceived stress status in older men in both the unadjusted or adjusted models. However, older women with hypovitaminosis D were twice as likely to perceive high stress in the fully adjusted model.

Previous studies have examined the relationship between hypovitaminosis D and psychological health status, mainly depression, and cognitive performance [13,14,15,16,17,18,19,20,21,22,23]. However, no previous studies have assessed the relationship between hypovitaminosis D and stress perception. Lower circulating vitamin D levels were associated with depressive symptoms, and higher serum 25(OH)D levels were associated with a reduced risk of depressive symptoms in several cross-sectional studies and a prospective study [13,14,15,16]. A lower serum 25(OH)D concentration has been found to be associated with lower cognitive function in several cross-sectional studies and a prospective study [17,19,20,21,22,23].

In contrast to the above studies, other studies have failed to find an independent association between hypovitaminosis D and psychological health status, mostly depression, and cognitive function. In an observational study of older Chinese men, an inverse relationship between vitamin D and baseline depression was observed; however, no association was observed after four years [36]. In cross-sectional studies, serum 25(OH)D levels were not significantly associated with depression [37,38], and a protective effect of vitamin D in the pathogenesis of depression was not observed in urban employees [39]. In the elderly, the relationship between 25(OH)D levels and overall cognitive function remains unknown [40]. Moreover, elderly subjects who scored highest on the cognitive function test were in the lowest quintiles of vitamin D concentrations in the NHANES III study [40].

The precise mechanism by which hypovitaminosis D causes high stress perception remains unclear. Different mechanisms through which vitamin D may potentially affect brain function have been suggested [9,10,41]. First, vitamin D may have a direct neuroregulatory activity. Vitamin D receptors and 25(OH)D3 1α-hydroxylase, the cytochrome P450 that catalyzes the hydroxylation of calcidiol to calcitriol, the bioactive form of vitamin D, are widely distributed throughout the CNS [42]. Second, vitamin D controls the expression of meaningful neurotrophic factors that affect neurotransmission and synaptic plasticity [10]. Third, vitamin D has been demonstrated to be neuroprotective, most importantly by inducing the synthesis of calcium-binding proteins or antioxidant mechanisms [9,10,41]. Fourth, the immunomodulatory activity of vitamin D has been associated with the role of inflammation in psychosocial stress. Vitamin D has been revealed to down-regulate inflammatory mediators such as nuclear factor κB, which have been associated with sickness behavior, psychosocial stress, and depression [10,31]. More importantly, the hippocampus is one of the brain regions with high concentrations of vitamin D receptors [43], and vitamin D has been shown to play a critical role in hippocampal cell survival through its neuroprotective effects [44].

A hallmark of the stress response is the activation of the autonomic nervous system and HPA axis [45]. In a cross-sectional study of healthy adults aged >20 years in Korea, lower 25(OH)D levels were associated with lower heart rate variability due to the autonomic nervous system dysfunction [46]. The hypothalamus and brain stem are essential for autonomic and neuroendocrine responses to stressors, and the hippocampus also plays a role in shutting off the HPA stress response [47,48]. Activation of the HPA axis produces glucocorticoids and affects glucocorticoid access to the brain [49]. A number of hormones such as insulin-like growth factor 1, ghrelin, and leptin have been shown to affect the hippocampus [50]. Insulin-like growth factor 1 is a member of the growth hormone family. Growth hormone is expressed in the hippocampus where it is upregulated by acute stress; in women, the expression of growth hormone is correlated with estradiol levels [51]. Future studies are required to identify the exact mechanism through which hypovitaminosis D causes high stress perception. In addition, the role of stress in the relationship between hypovitaminosis D and other psychologic disorders should be evaluated in further research.

The current analysis was stratified by men and women to examine the interaction between sex and hypovitaminosis D because mean vitamin D concentrations and the prevalence of hypovitaminosis D were significantly different between men and women. Sex differences in the relationship between hypovitaminosis D and stress perception could be attributable to different factors. One explanation for the non-significant association between hypovitaminosis D and stress perception in men could be the small number of men with hypovitaminosis D. Nashold et al. (2009) found that estradiol mediates the enhancement of CNS vitamin D3 receptor transcription and function [52]. Correale et al. (2010) identified a stronger immunomodulatory effect of vitamin D in women than in men, suggesting a synergy between vitamin D and estradiol, which upregulates growth hormone expression in the hippocampus by stress [53]. It could be hypothesized that a similar mechanism may be responsible for the relationship between hypovitaminosis D and perception of stress in women. Women tend to use products for protection against sunlight (i.e., sun exposure-blocking clothing, sunblock creams, sunglasses, and hats) more often than men, which may result in sex differences. In the present study, women were older than men. The hormonal status of older women could have induced a poor health status, including hypovitaminosis D. These factors should be considered as possible causes of hypovitaminosis D in women [54].

The present study had both strengths and limitations. A major strength of our study was the large-scale nationally representative sampling of the general Korean elderly population, and the investigation of the association between hypovitaminosis D and stress perception after stratification by sex. We also controlled for various potential confounding factors known to be associated with serum vitamin D levels and stress. Moreover, to our knowledge, this was the first study in which hypovitaminosis D was found to be related to stress perception in older Koreans. However, some limitations should be considered. First, this study was performed as a cross-sectional study in which all data were collected simultaneously; therefore, the causative relationship between serum 25(OH)D and stress perception is uncertain. Second, perceived stress evaluation was restricted to a single question (i.e., “During usual life activities, how much do you feel stressed?”). Therefore, we were unable to distinguish the details of the stress from a physiological perspective, including the maintenance of homeostasis, and we did not use a scale with known psychometric properties [55]. Therefore, understanding the results can be challenging [25]. Third, the sex-specific biological mechanism through which hypovitaminosis D correlates to high stress perception remains unclear. Finally, other confounding factors associated with vitamin D levels, such as seasonality of blood sampling and sunlight exposure, should be considered in future studies.

Hypovitaminosis D is highly prevalent among the elderly worldwide [1]. In the future, the prevention of hypovitaminosis D may become a strategy to prevent high stress perception [56] and its harmful health outcomes [41] among the elderly. This possibility is supported by the fact that sufficient vitamin D can directly and indirectly affect CNS by neuroregulatory, neurotrophic, neuroprotective, and immunomodulatory activity [9,10,31,41,42]. Fortunately, hypovitaminosis D can be treated by consuming vitamin D rich food, using dietary supplements, and increasing sun exposure [8,57]. Furthermore, vitamin D levels can be easily screened and effectively treated. We suggest screening vitamin D levels periodically among older patients and providing supplements if appropriate.

## 5. Conclusions

In summary, hypovitaminosis D was found to be significantly associated with perceived stress status after adjusting for possible confounders in elderly Korean women. Further studies are required to replicate this finding through appropriately designed, randomized, and controlled trials. Moreover, future studies are required to identify factors responsible for sex-specific biological mechanisms responsible for the correlation between hypovitaminosis D and high stress perception.

## Figures and Tables

**Table 1:** General characteristics of study participants by sex.

Variables	Total (*n* = 1393)	Men (*n* = 613)	Women (*n* = 780)	*p*-Value	Effect Size
Age (years)	71.35 ± 0.19	70.86 ± 0.23	71.75 ± 0.24	0.003	0.001
Body mass index (kg/m^2^)	24.05 ± 0.11	23.49 ± 0.14	24.49 ± 0.15	<0.001	0.024
Waist circumference (cm)	84.06 ± 0.33	85.09 ± 0.42	83.22 ± 0.43	0.001	0.012
Low household income	77.7 ± 1.7 (1052)	72.9 ± 2.5 (443)	81.5 ± 1.7 (609)	<0.001	0.69
Urban residence	79.9 ± 3.7 (962)	72.5 ± 3.7 (437)	67.9 ± 4.1 (525)	0.056	0.051
Current smoker	12.3 ± 1.0 (164)	23.1 ± 1.9 (141)	3.6 ± 0.8 (23)	<0.001	0.323
Alcohol drinker	38.2 ± 1.6 (516)	60.7 ± 2.4 (364)	19.9 ± 1.7 (152)	<0.001	0.413
Regular exercise	40.6 ± 1.6 (576)	48.7 ± 2.4 (306)	34.1 ± 2.1 (270)	<0.001	0.153
Alone at home	16.8 ± 1.1 (271)	6.9 ± 1.1 (47)	24.8 ± 1.7 (224)	<0.001	0.263
Hypertension	87.0 ± 1.2 (740)	82.6 ± 2.1 (287)	90.4 ± 1.4 (453)	0.002	0.107
Diabetes mellitus	55.8 ± 2.7 (244)	49.1 ± 4.2 (112)	62.4 ± 3.4 (132)	0.019	0.070
Dyslipidemia	65.0 ± 2.5 (310)	52.8 ± 4.2 (103)	74.4 ± 2.8 (207)	<0.001	0.201
Energy intake (kJ/day)	7096 ± 114.72	8267 ± 155.72	6150 ± 114.13	<0.001	0.104
Sleep duration (h/day)	6.79 ± 0.23	6.94 ± 0.12	6.68 ± 0.36	0.423	0.001
25-Hydroxyvitamin D (nmol/L)	46.65 ± 0.72	47.90 ± 0.85	45.64 ± 0.92	0.032	0.004
Hypovitaminosis D *	15.8 ± 1.4 (207)	12.6 ± 1.8 (69)	18.3 ± 1.7 (138)	0.013	0.096
High stress ^†^	19.6 ± 1.4 (278)	11.2 ± 1.5 (74)	26.4 ± 2.1 (204)	<0.001	0.177

Data are presented as estimated means ± standard errors (%; unweighted number) for categorical variables or estimated means ± standard errors for continuous variables. *p*-values were obtained by Pearson’s chi-squared test for categorical variables or general linear model analysis for continuous variables using a complex sample design. Effect size was determined by partial eta squared or Cramer’s V. * Hypovitaminosis D was defined as a 25-Hydroxyvitamin D level < 30 nmol/L; ^†^ High stress (score of 3 or 4) was classified using a self-reported questionnaire using the following question: “During usual life activities, how much do you feel stressed?” (1 = almost no stress, 2 = a little stress, 3 = much stress, 4 = very much stress).

**Table 2:** General characteristics of men according to vitamin D levels.

Variables	Vitamin D Sufficiency * (*n* = 544)	Hypovitaminosis D * (*n* = 69)	*p*-Value	Effect Size
Age (years)	70.78 ± 0.24	71.42 ± 0.54	0.272	0.001
Body mass index (kg/m^2^)	23.42 ± 0.14	24.04 ± 0.36	0.096	0.004
Waist circumference (cm)	84.83 ± 0.44	86.87 ± 1.13	0.088	0.006
Low household income	72.9 ± 2.6 (393)	72.5 ± 6.0 (50)	0.940	0.003
Urban residence	70.3 ± 4.0 (378)	88.0 ± 4.4 (59)	0.007	0.108
Current smoker	21.4 ± 2.0 (121)	34.9 ± 6.6 (20)	0.034	0.033
Alcohol drinker	60.0 ± 2.6 (319)	65.3 ± 6.3 (45)	0.472	0.028
Regular exercise	50.0 ± 2.5 (264)	60.6 ± 5.6 (43)	0.086	0.074
Alone at home	6.5 ± 1.1 (39)	9.6 ± 3.7 (8)	0.359	0.048
Hypertension	83.5 ± 2.0 (255)	76.9 ± 6.4 (32)	0.301	0.069
Diabetes mellitus	48.0 ± 4.0 (94)	55.4 ± 9.6 (18)	0.476	0.043
Dyslipidemia	53.0 ± 4.2 (90)	51.5 ± 10.6 (13)	0.889	0.039
Energy intake (kJ/day)	8363 ± 172.84	4941 ± 309.11	0.028	0.009
Sleep duration (h/day)	6.93 ± 0.10	7.01 ± 0.66	0.903	0.006
25-Hydroxyvitamin D (nmol/L)	51.17 ± 0.72	24.38 ± 0.70	<0.001	0.295
High stress ^†^	11.2 ± 1.7 (63)	11.6 ± 3.7 (11)	0.912	0.036

Data are presented as estimated means ± standard errors (%; unweighted number) for categorical variables or estimated means ± standard errors for continuous variables. *p*-values were obtained by Pearson’s chi-squared test for categorical variables or general linear model analysis for continuous variables using a complex sample design. Effect size was determined by partial eta squared or Cramer’s V. * Vitamin D sufficiency was defined as a 25-Hydroxyvitamin D level ≥ 30 nmol/L. Hypovitaminosis D was defined as a 25-Hydroxyvitamin D level < 30 nmol/L; ^†^ High stress (score of 3 or 4) was classified using a self-reported questionnaire using the following question: “During usual life activities, how much do you feel stressed?” (1 = almost no stress, 2 = a little stress, 3 = much stress, 4 = very much stress).

**Table 3:** General characteristics of women according to vitamin D levels.

Variables	Vitamin D Sufficiency * (*n* = 642)	Hypovitaminosis D * (*n* = 138)	*p*-Value	Effect Size
Age (years)	71.99 ± 0.25	70.69 ± 0.56	<0.001	0.003
Body mass index (kg/m^2^)	24.37 ± 0.17	25.02 ± 0.35	0.111	0.001
Waist circumference (cm)	83.03 ± 0.48	84.08 ± 0.88	0.277	0.000
Low household income	81.8 ± 1.9 (501)	80.2 ± 3.8 (108)	0.708	0.012
Urban residence	66.3 ± 4.2 (417)	75.1 ± 6.0 (108)	0.141	0.120
Current smoker	3.6 ± 0.9 (18)	3.9 ± 1.8 (53)	0.877	0.017
Alcohol drinker	20.1 ± 1.9 (126)	19.3 ± 4.2 (26)	0.868	0.010
Regular exercise	64.4 ± 2.4 (415)	72.7 ± 4.7 (94)	0.142	0.026
Alone in home	26.2 ± 1.9 (192)	18.3 ± 3.6 (32)	0.074	0.054
Hypertension	90.1 ± 1.3 (370)	91.6 ± 2.8 (83)	0.638	0.010
Diabetes mellitus	63.8 ± 3.6 (110)	56.8 ± 8.6 (22)	0.476	0.041
Dyslipidemia	74.9 ± 2.8 (160)	73.0 ± 6.3 (47)	0.783	0.013
Energy intake (kJ/day)	6163 ± 130.58	6091 ± 183.59	0.748	0.000
Sleep duration (h/day)	6.49 ± 0.32	7.53 ± 1.35	0.454	0.000
25-Hydroxyvitamin D (nmol/L)	50.12 ± 0.85	25.10 ± 0.37	<0.001	0.348
High stress ^†^	25.4 ± 2.4 (163)	31.1 ± 5.0 (41)	0.305	0.025

Data are presented as estimated means ± standard errors (%; unweighted number) for categorical variables or estimated means ± standard errors for continuous variables. *p*-values were obtained by Pearson’s chi-squared test for categorical variables or general linear model analysis for continuous variables using a complex sample design. Effect size was determined by partial eta squared or Cramer’s V. * Vitamin D sufficiency was defined as a 25-Hydroxyvitamin D level ≥ 30 nmol/L. Hypovitaminosis D was defined as a 25-Hydroxyvitamin D level < 30 nmol/L. **^†^** High stress (score of 3 or 4) was classified using a self-reported questionnaire using the following question: “During usual life activities, how much do you feel stressed?” (1 = almost no stress, 2 = a little stress, 3 = much stress, 4 = very much stress).

**Table 4:** Odds ratios (95% confidence intervals) of perceived stress status according to vitamin D levels.

Sex	Model	Vitamin D Sufficiency (≥30 nmol/L)	Hypovitaminosis D (<30 nmol/L)	*p*-Value
Men	Unadjusted	1.00 (Reference)	1.045 (0.481–2.270)	0.912
	Model 1	1.00 (Reference)	1.067 (0.491–2.323)	0.869
	Model 2	1.00 (Reference)	1.126 (0.521–2.433)	0.762
	Model 3	1.00 (Reference)	0.829 (0.172–4.002)	0.815
Women	Unadjusted	1.00 (Reference)	1.329 (0.771–2.290)	0.305
	Model 1	1.00 (Reference)	1.297 (0.762–2.206)	0.337
	Model 2	1.00 (Reference)	1.268 (0.744–2.160)	0.383
	Model 3	1.00 (Reference)	2.739 (1.107–6.774)	0.029

Model 1: Adjusted for age; Model 2: Additionally adjusted for body mass index and waist circumference; Model 3: Additionally adjusted for smoking status, alcohol consumption, physical activity, history of hypertension, diabetes mellitus, and dyslipidemia; *p*-values were obtained using multiple logistic regression analysis in a complex sample design.
